# Knowledge Regarding Mechanical Ventilation and Practice of Ventilatory Care among Nurses Working in Intensive Care Units in Selected Governmental Hospitals in Addis Ababa, Ethiopia: A Descriptive Cross-Sectional Study

**DOI:** 10.1155/2023/4977612

**Published:** 2023-02-13

**Authors:** Kedir Abdureman Hassen, Micheal Alemayehu Nemera, Andualem Wubetie Aniley, Ararso Baru Olani, Sofoniyas Getaneh Bedane

**Affiliations:** ^1^Department of Nursing, College of Health Sciences, Ambo University, Ambo, Ethiopia; ^2^Department of Emergency and Critical Care, Tirunesh Beijing General Hospital, Addis Ababa, Ethiopia; ^3^Department of Emergency Medicine, College of Health Sciences, Addis Ababa University, Addis Ababa, Ethiopia; ^4^College of Medicine and Health Sciences, Arba Minch University, Arba Minch, Ethiopia; ^5^Department of Anesthesiology and Critical Care, Saint Peter Specialized Hospital, Addis Ababa, Ethiopia

## Abstract

**Introduction:**

Mechanical ventilation (MV) is a backbone and major supportive modality in intensive care units (ICUs) even though it has side effects and complications. Knowledge of nurses about mechanical ventilators and good practice of nursing care for the ventilated patient plays a crucial role in improving the effectiveness of mechanical ventilation, preventing harm, and optimizing the patient outcome. This study intended to assess the knowledge regarding MV and the practice of ventilator care among nurses working in the ICU.

**Method:**

A descriptive cross-sectional study design was conducted. All nurses working in the intensive care unit of selected governmental hospitals were included in the study. The data were collected from March 1 to 30, 2021 with structured and pretested self-administered questionnaires. The collected data were evaluated with SPSS version 26 software. The variables, which have an independent association with poor outcomes, were identified based on OR, with 95% CI and a *p* value less than 0.05.

**Results:**

Of 146 nurses who participated in the study, 51.4% were males. About 71.4% had a BSc in nursing and 57.5% of them had training related to MV. More than half (51.4%) of nurses had poor knowledge regarding MV and the majority (58.9%) of them had poor practice in ventilatory care. The educational level (AOR, 5.1; 95% CI, 1.190–22.002) was positively associated with knowledge. Likewise, the educational level (AOR 5.0 (1.011–24.971)) and work experience (AOR 4.543 (1.430–14.435)) were positively associated with the practice of nurses.

**Conclusions:**

Knowledge regarding mechanical ventilators and the practice of ventilatory care among nurses in the selected public hospitals was poor. The educational levels were found statistically associated with both the knowledge and practice of nurses. To improve nursing care offered for MV patients, upgrading the educational level of intensive care nurses plays a vital role.

## 1. Introduction

One-third of the patients admitted to the intensive care unit (ICU) in the world need mechanical ventilation (MV) treatment. Mechanical ventilator support is one of the main indications for admission to the intensive care unit (ICU). The continuous development of mechanical ventilators and their clinical use have been critical factors for the development and growth of critical care and respiratory professions [1, 2].

A mechanical ventilator is a machine that is essential to support patients to breathe when they are unable to ventilate and oxygenate on their own due to critical illness or having surgery. Patients are connected to a ventilator by a hollow tube that goes from their mouth to their trachea, thereby providing the function of respiratory muscles. In the meantime, the patient remains on the ventilator until they can breathe independently [3].

Even though mechanical ventilation can save lives, it also carries several side effects and complications such as asynchrony, auto-PEEP, barotrauma, hemodynamic compromise, nosocomial infection, anxiety/stress/sleep deprivation, ulcers/gastritis malnutrition, and muscle deconditioning/vent dependence. Increased intrathoracic pressure may lead to systemic edema because the venous return is decreased [4]. This is often with prolonged mechanical ventilation, increased mortality, prolonged hospital stay, and high cost to the patient [5]. In developed countries, 2 to 3 million intensive care unit patients receive invasive mechanical ventilation per year at estimated costs of 15–27 billion dollars [6].

The management of critically ill patients has become increasingly important in modern medical and nursing systems. Critical care nurses play a crucial role in improving the effectiveness of mechanical ventilation, preventing harm, and optimizing patient outcomes. The skills and knowledge of health teams regarding the care of a patient on a mechanical ventilator and patients' clinical status enable them to fine-tune ventilator settings to maximize the benefits of ventilator support while minimizing complications [5, 6].

Knowledge of nurses about the functions and limitations of ventilator modes, causes of distress, dyssynchrony with ventilator, and appropriate management enable them to provide high-quality centered care [7, 8]. As nurses are the first-line manager challenged with patients' and ventilators' problems, it is crucial to recognize the problems such as respiratory distress, dyspnea, and increased work of breathing, and the actions to be taken to solve these problems. So, the nurses who provide care for ventilated patients must understand the basic ventilator support including ventilator mode, setting, and alarms. It is also important to be skilled in promptly identifying and managing common patient and ventilator-related problems to provide optimal patient-centered care and prevent complications [9].

Studies have shown that ICU nurses' knowledge of mechanical ventilation is globally poor. A study conducted in South Africa has reported that even in high-income countries where nursing education is advanced, the levels of knowledge on mechanical ventilation are not perfect [8].

Therefore, this study is anticipated to be crucial, as it will intend to discover more data regarding the levels of knowledge and practice of nurses working in the ICU on mechanical ventilator utilization.

## 2. Methods

### 2.1. Study Design

A cross-sectional study design was conducted to assess the knowledge regarding mechanical ventilators and the practice of ventilatory care among nurses working in the intensive care unit.

### 2.2. Study Setting and Period

The study was conducted in Addis Ababa, which is the capital city of Ethiopia. The town is the seat of the Africa Union and different continental organizations. The city consists of 34 private hospitals and 14 public hospitals [7, 9]. The study was conducted in purposively selected five intensive care units of selected public hospitals. These are Tikur Anbessa, St. Peters, AaBET, Yekatit12, and Menelik II Hospitals where well-organized ICU services with a full mechanical ventilator are provided. The study was conducted from March to April 2021 among intensive care nurses at selected public hospitals.

### 2.3. Study Participants

The source population was all nurses who were working in intensive care units of the selected governmental hospitals in Addis Ababa, Ethiopia. Eligibility criteria included having experience in the intensive care unit at least for six months and having consent to participate in the study. Thus, this study included participants from five selected public hospitals.

### 2.4. Sample Size Determination

All nurses working in the ICU of selected hospitals who fulfilled the inclusion criteria were taken as the sample size of the study.

### 2.5. Variables

#### 2.5.1. Dependent Variables

The dependent variable in this study was the knowledge of nurses regarding mechanical ventilators and the practice of ventilatory care.

#### 2.5.2. Independent Variable

Independent variables of the study included sociodemographic variables such as age, sex, educational level, work experience, and training.

### 2.6. Data Collection Techniques and Instrument

A self-administered structured questionnaire was utilized to collect information from each participant. The English version of the questionnaire was adopted from different literature based on elements intended to study. The questionnaires have three parts; part one sociodemographic factors, part two knowledge assessment consisting of 16 items, and part three practice assessment questions consisting of 25 items.

### 2.7. Data Quality Assurance

The questionnaire was pretested before data collection and the possible corrections were made. Besides, two days of training were provided for the data collectors and supervisors. The data collectors were health professionals, who hold a Bachelor of Science in Emergency and Critical care nursing, for better understanding and interpretation of the questionnaire. Additionally, a pilot test was conducted but the results were excluded from the actual study. Based on the feedback from the pilot study, immediate corrective measures were taken. Furthermore, continuous and close supervision of the data collecting procedures, proper categorization, and coding of the data were conducted. The Principal Investigators and the supervisors checked the completeness and consistency of data daily.

### 2.8. Data Entry and Analysis Procedure

The collected data were entered with Epi-Data version 4.6 and analyzed with SPSS version 26.0 software. Participants' characteristics were examined using frequency, percentage distribution, mean, and standard deviation. The binary logistic regression analysis was conducted and all independent variables with a *p* value less than 0.25 were included in a multivariable logistic regression model to identify factors associated with knowledge regarding MV and practices of ventilatory care. The level of association and statistical significance was measured using an odds ratio with a 95% confidence interval. The statistical significance was set at *p* value <0.05.

### 2.9. Ethical Consideration

Permission to conduct the research was obtained from AAU, CHS research standards, Ethics Committee, and Addis Ababa public health and emergency directorate. The research purpose, benefits, and procedures were explained to each potential respondent. The respondents gave an informed written and oral consent and any respondent seeking further clarification was provided. The information that the respondents provided during the study was kept confidential.

## 3. Results

### 3.1. Sociodemographic Characteristics

The average age of the participant was 29.84 years (SD = 4.95). Approximately half of the participants were male 71 (51.4%), and the majority 104 (71.2%) had a bachelor's degree. Concerning training, most (57.5%) of the respondents had training related to a mechanical ventilator (Table 1).

### 3.2. Description of the Knowledge of the ICU Nurses Related to Mechanical Ventilation

**Figure 1:**
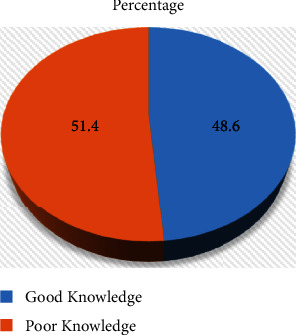
Level of knowledge regarding mechanical ventilators among nurses working in the ICU of selected government hospitals in Addis Ababa, Ethiopia, 2021 (*N* = 146).

More than half of the participants, that is, 75 (51.4%) had poor and 71 (48.6%) had good knowledge regarding mechanical ventilators (Figure 1).

### 3.3. Item-Wise Response of Nurses Regarding Knowledge Related to Mechanical Ventilation

About 112 (76.7%) nurses did not know the amount of PaO_2_ in the initiation of a mechanical ventilator, 95 (61.5%) of them were not aware of the critical amount of respiratory rate, and nearly half 75 (51.4%) of them did not know critical pH values to initiate ventilation. Regarding the parameters of the mechanical ventilator, the majority 93 (63.7%) of the participant expand the pressures-targeted ventilation mode while nearly two-thirds of 99 (68.8%) nurses were not familiar with the volume-targeted ventilation mode. Almost half 75 (51.4%) of the participants could explain FIO_2,_ the majority 100 (68.5%) of them were familiar with PEEP and its function, and 78 (53.4) of nurses could not expand the tidal volume. In the context of alarms, nearly two-thirds of 96 (65.8%) nurses were aware of the cause of high-pressure alarms, and more than half of 83 (56.8) participants did not know the cause of low-pressure alarms. Ninety-two (63%) of nurses could not recognize whether the ventilator flow rate setting matched patient inspiratory efforts or not. More than half of 86 (58.9%) participants did not know the changes that should be made to match the inspiratory demand of the patient to breathe fast. Most 79 (54.1) nurses were not aware of the potential results of alarm silencing once or repeatedly. Nearly half 76 (52.1%) of nurses were not familiar with signs and symptoms indicating a patient who is not ready to be weaned, majority 83 (56.8) of them were not familiar with the correct sequence of weaning, and 84 (57.5) of them were familiar with extubation (Table 2).

### 3.4. Description of the Practice of the ICU Nurses Related to Ventilator Care

**Figure 2:**
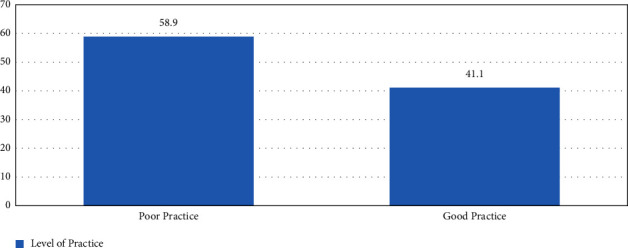
Level of practice regarding mechanical ventilator care among nurses working in the ICU of selected government hospitals in Addis Ababa, Ethiopia 2021 (*N* = 146).

Out of 146, the majority 86 (58.9%) nurses had poor and 60 (41.1%) had good practice related to ventilator care (Figure 2).

### 3.5. The Item-Wise Practice of Nurses Related to Ventilator Care

Practice related to ventilator care revealed that most of the 134 (90.4%) participants preoxygenated the patient before suctioning, 61 (41.8%) of them did suction as per needed, and more than two-thirds of 106 (72.6%) nurses instill normal saline before suctioning. The Majority of 117 (80.1%) nurses check the level of the endotracheal tube and 54 (37%) of them change the position of ETT every 24 h (Table 3).

Regarding cuff pressure checking, 54 (37%) of them checked the cuff pressure every 6–12 h. The manual (palpation) method of cuff pressure monitoring mechanism was majorly 101 (69.2%) used to monitor cuff pressure. The cuff pressure measurement (CPM) and minimal leak test (MLT) were other methods used to monitor cuff pressure by 33 (22.6%) and 22 (15.1%) nurses, respectively.

Nearly half of 74 (50.7%) participants recognized the complications of ETT. Study participants employed various eye care methods. Among these, the majority 105 (71.9%) of the participants were wiping eyes from the inner canthus to out, 59 (40.4%) taped their eyes shut, and 46 (31.5%) of them used teardrop as an eye care method. A total of 79 (54.1%) of these individuals practice oral hygiene once a day, whereas 52 (35.6%) practice it twice a day. Additionally, two-thirds of the participants, 90 (61.6%), used chlorhexidine to clean patients' mouths, while 30 (20.5%) of them used sodium bicarbonate.

More than half of 78 (53.4%) participants recognized anxiety-related findings on a mechanical ventilated patient. Various methods have been used to relieve the anxiety of the patient receiving ventilation. Most 106 (69.2%) relaxed the patients by talking to them, 52 (35.6%) helped the patients to express themselves in writing, and 72 (49.2%) helped them by contacting family members. More than two-thirds of 101 (69.2%) participants were not following the ventilator care bundle.

### 3.6. Factors Associated with Knowledge of Nurses Working in ICU Regarding Mechanical Ventilation

In multivariate logistic regression analysis, the educational level was found to be a statistically significant positive association with the knowledge level at a *p* value of less than 0.05, but the other variables were not found to be statistically significant. The odds of those who had a diploma were 5.116 times more likely (AOR, 5.116; 95% CI, 1.190–22.002) to have poor knowledge than those who had an MSc (Table 4).

### 3.7. Factors Associated with Nurses' Level of Practice toward Caring for Patients on Mechanical Ventilation Support

Regarding the factors associated with the level of practice educational level and work experience had a significant positive association at a *p* value less than 0.05.

The probability of those who had a diploma was 5.024 times (AOR 5.024 (1.011–24.971)) less likely to have a good practice than those who had MSc. On the other hand, those who had an experience of more than ten years were 4.543 times (AOR 4.543 (1.430–14.435) more probable to have good practice than those who had an experience of 1–5 years (Table 5).

## 4. Discussion

Nurses play a crucial role in the management of patients on mechanical ventilators. There is no doubt that nurses must have in-depth scientific knowledge and demonstrate evidence-based practice in providing care to mechanically ventilated patients. So, this study aimed to determine the knowledge level of nurses working in ICU regarding MV and their practice level towards caring for a patient on MV support.

In this study, more than half (51.4%) of the study participants were male and a majority (58.9%) of the nurses were in the age group of (20–29) years old. This finding was similar to a study conducted in Iraq and India, which showed that 58% and 64% of the participant were male and the majority 55% and 58% of nurses were in the age group below 29 [4, 8].

Regarding the training-level majority (57.8%) of ICU nurses had training related to a mechanical ventilator. However, the study conducted in Egypt, Iraq, and Turkey showed a lower level of trained nurses in their ICUs, which are 36%, 56%, and 30%, respectively [5, 8, 10]. This difference might be due to this research conducted where considerable attention is given to critical care.

More than half (51.4%) of the study participants had poor knowledge of mechanical ventilation. This finding agrees with the study conducted in southern India in which 53.5% of the nurses had poor knowledge [11]. On the other hand, the study conducted in Sri Lanka and Eastern India showed a higher knowledge level among nurses, which are 73% and 69.7%, and had good knowledge regarding mechanical ventilation [6, 12]. This discrepancy could be attributed to the sociodemographic difference and the difference in the type of ICU.

The majority (58.6%) of nurses had poor practice regarding the care of a patient on a mechanical ventilator. These results are consistent with other research which found that the majority (93%) of ICU nurses had poor practice regarding the care of a patient on a mechanical ventilator [11]. However, the study from Sri Lanka revealed that the majority (57.8%) of the nurses had good practice regarding the care of ventilated patients [13]. This inconsistency may be due to the difference in the tool used to assess the practice and another possible explanation for this is the difference in the study setting.

A significant relationship was found between nurses' educational level and their knowledge in this study. Nurses who had diplomas were found to have less knowledge about mechanical ventilators than BSc and MSc holders. This finding is consistent with that of the study in Sri Lanka, in which the educational level was statistically associated with nurses' knowledge level. Similarly, those who had diplomas had less knowledge than BSc and MSc holders [13].

The educational level and work experience of nurses were significantly associated with the practice of nurses. Our finding is different from the study conducted in Egypt, which showed job position as a factor that affects the practice level. These results are probably related to the socioeconomic differences between study settings.

### 4.1. Limitations of the Study

The results of this study are subject to some limitations. Due to the cross-sectional nature of the study, no direct intervention or direct observation of the study participant was conducted. The limited number of nurses is another limitation. Although this study has limitations, it also has a strength. It is the first study that attempts to assess the level of knowledge regarding the MV and ventilatory care practice among nurses working in the ICU of selected government hospitals in Addis Ababa, possibly in Ethiopia. It also tried to incorporate most of the pertinent components of ventilator care.

### 4.2. Generalizability

The potential generalizability of the evidence generated by this study to other settings should be considered in view of the study setting, context, methods, and limitations described in this study.

## 5. Conclusion

Knowledge regarding mechanical ventilators and the practice of ventilatory care among nurses in the selected public hospitals was poor. The educational level was found to be statistically associated with both the knowledge and practice of nurses. Additionally, years of experience were also significantly associated with the level of practice. To improve nursing care offered for mechanically ventilated patients, upgrading the educational level of intensive care nurses plays a vital role.

## Figures and Tables

**Table 1:** Sociodemographic characteristics of nurses working in the ICU of selected hospitals in Addis Ababa, 2021.

Variables	Categories	Frequency (*N* = 146)	Percent (%)
Gender	Male	75	51.4
	Female	71	48.6

Age	20–29 yrs	86	58.9
	30–39 yrs	54	37
	≥40 yrs	6	4.1

Educational level	Diploma	18	12.3
	BSc nurse	104	71.2
	MSc nurse	12	8.2
	Critical care nurse	12	8.2

Participating in training related to a mechanical ventilator	Yes	84	57.5
	No	62	42.5

Years of working experience	<1 yrs	12	8.2
	1–5 yrs	84	57.5
	6–10 yrs	38	26
	>10 yrs	12	8.2

Marital status	Single	83	56.8
	Married	61	41.8
	Divorced	2	1.4

**Table 2:** Knowledge of nurses working in intensive care units regarding mechanical ventilators at the selected government hospitals in Addis Ababa, Ethiopia 2021 (*N* = 146).

Knowledge regarding MV	Yes *N* = 146 (%)	No *N* = 146 (%)
Knowing the amount of pao_2_ in the initiation of a mechanical ventilator	34 (23.3)	112 (76.7)
Knowing the critical amount of respiratory rate in the initiation of a mechanical ventilator	51 (34.9)	95 (61.5)
Knowing the critical pH values in the indication of mechanical ventilator	71 (48.6)	75 (51.4)
Knowing volume-targeted mechanical ventilator mode	47 (32.2)	99 (68.8)
Knowing pressures-targeted mechanical ventilator mode	93 (63.7)	53 (36.3)
Knowing the explanation of the term FIO_2_	75 (51.4)	71 (48.6)
Knowing the term PEEP and its functions	100 (68.5)	46 (31.5)
Knowing the term tidal volume	68 (46.6)	78 (53.4)
Knowing the cause of high-pressure alarms	96 (65.8)	50 (34.2)
Knowing the cause of low-pressure alarm	63 (43.2)	83 (56.8)
Knowing the observations that indicate the ventilator flow rate setting is matched with patient inspiratory efforts	54 (37.0)	92 (63.0)
Knowing the changes should make to match the inspiratory demand of the patient breathing fast	60 (41.1)	86 (58.9)
Knowing the potential results of alarm silencing once or repeatedly	67 (45.9)	79 (54.1)
Signs and symptoms indicate a patient who is not ready to be weaned	70 (47.9)	76 (52.1)
The correct sequence of weaning	63 (43.2)	83 (56.8)
Extubation of a patient	84 (57.5)	62 (42.5)

**Table 3:** Practice nurses working in ICU regarding ventilatory care at selected government hospitals in Addis Ababa, Ethiopia 2021 (*N* = 146).

Care practice	Yes	%	No	%
Endotracheal/tracheal tube suctioning (when required)	61	41.8	85	58.2
Checking of endotracheal tube level	117	80.1	29	19.9
Rotating endotracheal tube positions (every 24 hrs.)	54	37	92	63
Checking cuff pressures (every 6–12 hrs.)	54	37	92	63
Recognize complications of ETT	74	50.7	72	49.3
Giving routine normal saline instillation before suctioning	106	72.6	40	27.4
Preoxygenate the patient with 100% oxygen before suctioning	132	90.4	14	9.6
Following the anxiety of a patient on mechanical ventilation	107	73.3	39	26.7
Recognizing the anxiety finding of a patient on mechanical ventilation support	78	53.4	68	46.6
Informing the relatives of a ventilated patient	128	87.7	18	12.3
Following ventilator care bundle practice to prevent VAP	45	30.8	101	69.2

**Table 4:** Factors associated with the knowledge of nurses working in the ICU regarding mechanical ventilators (*N* = 146).

Variables		Knowledge status		COR (95% CI)	AOR (95% CI)	*p* value
		Good	Poor			
Gender	Male	34	41	1.312 (0.685–2.516)	1.263 (0.634–2.516)	0.506
	Female	37	34	1	1	

Age	20–27	25	25	0.778 (0.251–2.414)	0.321 (0.076–1.361)	0.123
	28–35	39	41	0.818 (0.277–2.409)	0.525 (0.155–1.779)	0.301
	≥36	7	9	1	1	

Educational level	Diploma	5	9	3.6 (0.902–14.376)	5.1 (1.190–22.002)	0.028^*∗*^
	BSc	50	58	2.320 (0.916–5.875)	2.747 (0.991–7.617)	0.052
	MSc	16	8	1	1	

Training on MV	Yes	37	47	1	1	
	No	34	28	0.648 (0.335–1.254)	0.629 (0.317–1.249)	0.185

Work experience	<1	5	7	1.4 (0.300–5.933)	2.3 (0.449–11.842)	0.316
	1–5	10	10	1.1 (0.145–2.977)	1.4 (0.468–4.288)	0.538
	6–10	36	49	0.9 (0.305–2.659)	0.880 (0.280–2.765)	0.826
	>10	20	18	1	1	

**Table 5:** Factors associated with the level of practice of nurses working in the ICU regarding mechanical ventilators (*N* = 146).

Variables		Practice status		COR (95% CI)	AOR (95% CI)	*p* value
		Good	Poor			
Gender	Male	35	40	1.610 (0.828–3.132)	0.544 (0.263–1.125)	0.100
	Female	25	46	1	1	

Age	20–27	22	28	0.990 (0.318–3.079)	0.340 (0.078–1.487)	0.340
	28–35	31	49	1.229 (0.415–3.639)	0.625 (0.183–2.141)	0.625
	≥36	7	9	1	1	

Educational level	Diploma	3	11	4.3 (0.959–19.579)	5.0 (1.011–24.971)	0.049^*∗*^
	BSc	44	64	1.719 (0.706–4.186)	2.516 (0.919–6.889)	0.073
	MSc	13	11	1	1	

Training on MV	Yes	37	47	1	1	
	No	23	39	1.335 (0.682–2.613)	1.371 (0.699–2.808)	0.389

Work experience	<1	7	5	1.1 (0.250–4.591)	1.8 (0.338–9.633)	0.489
	1–5	25	51	3.060 (1.109–8.440)	4.543 (1.430–14.435)	0.01^*∗*^
	6–10	16	22	2.1 (0.685–6.210)	2.2 (0.701–7.151)	0.174
	>10	12	8	1	1	

## Data Availability

The datasets used and/or analyzed during the current study are available without restriction.

## Abbreviations

CPM: Cuff pressure measurement
ETT: Endotracheal tube
FIO_2_: Fraction of inspired oxygen
ICU: Intensive care unit
MV: Mechanical ventilator
MLT: Minimal leak test
PEEP: Positive end-expiratory pressure
VAP: Ventilator-associated pneumonia.
